# NLRP3 Inflammasome: Activation and Regulation in Age-Related Macular Degeneration

**DOI:** 10.1155/2015/690243

**Published:** 2015-01-27

**Authors:** Jiangyuan Gao, Ruozhou Tom Liu, Sijia Cao, Jing Z. Cui, Aikun Wang, Eleanor To, Joanne A. Matsubara

**Affiliations:** Department of Ophthalmology and Visual Sciences, Faculty of Medicine, University of British Columbia, Vancouver, BC, Canada V5Z 3N9

## Abstract

Age-related macular degeneration (AMD) is the leading cause of legal blindness in the elderly in industrialized countries. AMD is a multifactorial disease influenced by both genetic and environmental risk factors. Progression of AMD is characterized by an increase in the number and size of drusen, extracellular deposits, which accumulate between the retinal pigment epithelium (RPE) and Bruch's membrane (BM) in outer retina. The major pathways associated with its pathogenesis include oxidative stress and inflammation in the early stages of AMD. Little is known about the interactions among these mechanisms that drive the transition from early to late stages of AMD, such as geographic atrophy (GA) or choroidal neovascularization (CNV). As part of the innate immune system, inflammasome activation has been identified in RPE cells and proposed to be a causal factor for RPE dysfunction and degeneration. Here, we will first review the classic model of inflammasome activation, then discuss the potentials of AMD-related factors to activate the inflammasome in both nonocular immune cells and RPE cells, and finally introduce several novel mechanisms for regulating the inflammasome activity.

## 1. Introduction

Age-related macular degeneration (AMD) is a neurodegenerative disease characterized by the deterioration of photoreceptors in the macula, a specialized region of the retina responsible for fine visual acuity that is required for tasks such as reading, facial recognition, and driving [1]. According to the World Health Organization, AMD currently ranks as the third global leading cause of blindness, second only to cataract and glaucoma [2]. However, among the elderly, AMD is the most common cause of irreversible vision loss in developed countries. Approximately 30–50 million individuals worldwide are afflicted with AMD. The economic costs for treatment and care of individuals who suffer vision loss from AMD are projected to be more than US$ 300 billion annually, a heavy toll that will significantly impact global social and public health systems and one that prompts an urgent need to decipher its underlying mechanisms [3].

**Figure 1:**
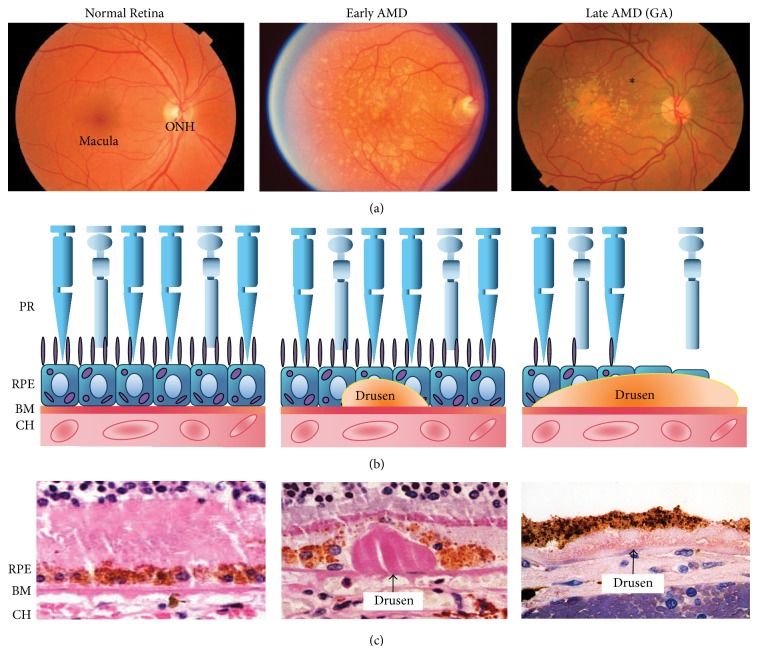
Clinical stages and signs of age-related macular degeneration. (a) Fundus photos demonstrate clinical features of AMD at different stages. Early AMD shows yellow extracellular drusen deposits surrounding macular area. Late AMD (GA) shows hypopigmentation or background darkening (∗) around drusen. A large number of drusen deposits are observed accumulated in the macular area. (b) Schematic diagram of drusen accumulation and RPE/photoreceptor degeneration from early to late stage AMD (GA). (c) Staining of human postmortem donor eye tissues depicting normal, early AMD, and late AMD. Arrows point to different forms of drusen: a large hard drusen in an early AMD eye and a diffuse, soft drusen in a late AMD (GA) eye. GA, geographic atrophy; ONH, optic nerve head; PR, photoreceptors; RPE, retinal pigment epithelium; BM, Bruch's membrane; CH, choroidal capillaries.

Being a complex disease, the pathogenesis and progression of AMD are influenced by a variety of risk factors. Among them, advanced chronologic aging is thought to be the strongest [4–6]. The prevalence of AMD steadily increases with age, affecting 2% of the population at age 40 and 25% by age 80 [7]. Besides aging, other risk factors such as cigarette smoking and diet also contribute to the development of the disease [8–11]. Clinically, early stages of AMD are defined by the presence of drusen, the extracellular deposits located between the retinal pigment epithelium (RPE) and Bruch's membrane (BM) (Figure 1). Despite the fact that early AMD is usually not associated with appreciable vision loss, the number and the size of drusen deposits serve as signs of disease progression [12]. When the disease progresses into the late stage, it takes one of two forms: geographic atrophy (GA), featured by confluent regions of RPE and photoreceptor degeneration, and choroidal neovascularization (CNV), characterized by the abnormal growth of leaky choroidal vessels invading retina. Initially considered a supporting cell in the outer retina, RPE are active in a wide range of biological processes that maintain local homeostasis. These processes include recycling components of the visual cycle, secreting trophic factors, controlling cross-epithelium transport, and maintaining the outer blood-retinal barrier [13, 14]. Central to AMD pathogenesis, the RPE undergoes significant changes in structure and function that predispose individuals to disease processes associated with AMD. Suggestive of an associated, and perhaps causal, role in RPE dysfunction is the finding that RPE cells overlying drusen appear swollen and vacuolated [15]. It is further proposed that the spontaneous release of drusen components during drusen regression in AMD development may result in RPE loss in GA [16].

## 2. NLRP3 Inflammasome

Recent advances have highlighted the essential role of immune processes in the development, progression, and treatment of AMD [1]. Both the innate and adaptive immune systems have been shown to contribute to AMD pathogenesis (for reviews, see [28, 29]). The innate immune system is an evolutionarily conserved system that constitutes the first line of defense against pathogens. Inflammasome activation is a key component of innate immunity, which when overactive has been linked with many human immune diseases [30–33]. The inflammasome is an intracellular, multiprotein complex whose molecular composition is stimulus dependent. The canonical inflammasome complexes are assembled around protein members of the nod-like receptor (NLRs) or HIN-200 protein families, converting the procaspase-1 zymogen into a catalytically active enzyme. The inflammasome family is further categorized based on the presence of an apoptosis-associated speck-like protein containing a caspase recruitment domain (ASC). The canonical inflammasome can be further categorized into ASC-dependent (NLRP3 and AIM2) and ASC-independent (NLRP1 and NLRC4) subtypes [34]. The noncanonical inflammasome complex with an, as yet, unknown structural composition is proposed to promote the activation of caspase-11 [35]. Despite the gap in knowledge of the structure of the noncanonical inflammasome, there is a wealth of evidence to firmly establish the mode of action for several canonical inflammasomes in the immune signaling pathway, especially the most widely studied NLRP3 inflammasome [36].

**Figure 2:**
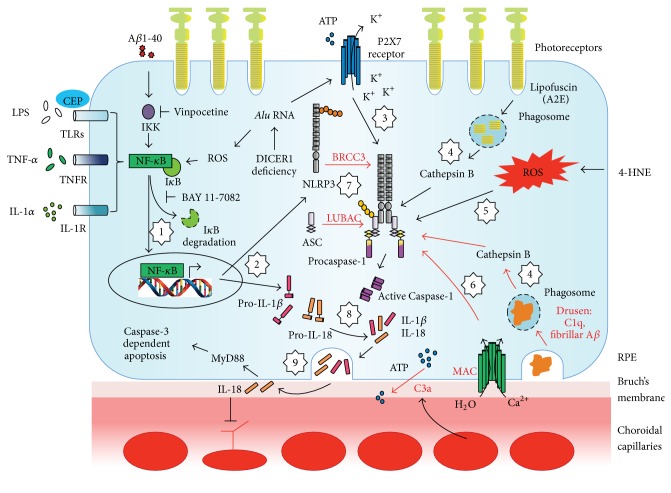
Current model of NLRP3 inflammasome activation in RPE. (1) Priming of the RPE by one of the following factors (LPS [17], TNF-*α* [17], IL-1*α* [17], CEP [18], and A*β*1-40 [19]) is needed in order to activate the NF-*κ*B pathway, which can be specifically blocked by vinpocetine or BAY 11-7082 [19]. Intriguingly, DICER1 deficiency induced* Alu* RNA accumulation has also been demonstrated to prime NF-*κ*B signaling, independent of toll-like receptors (TLRs) [20]. (2) Once the NF-*κ*B pathway is active, it promotes the transcription of NLRP3 and pro-IL-1*β*. (3–7) For the production of mature IL-1*β* and IL-18; separate inflammasome components are assembled as a multiprotein complex triggered by one of the following mechanisms: K^+^ efflux via P2X7 receptor activation in response to extracellular ATP accumulation or intracellular* Alu* RNA [20] (3); cytoplasmic cathepsin B release from destabilized phagolysosomes of lipofuscin/A2E [21] (4); ROS overproduction caused by 4-HNE [22] (5). Other NLRP3 inflammasome activation mechanisms that have been reported in immune cells but not validated in RPE cells are shown in red text and arrows. These include drusen components (C1q [18] and fibrillar A*β* [23]) induced lysosomal damage (4), C3a triggered ATP efflux [24], MAC formation [25] (6), BRCC3-mediated deubiquitylation [26], and LUBAC-mediated ubiquitylation [27] (7). (8) Successful assembly of NLRP3 inflammasome triggers autoproteolysis of procaspase-1 into active caspase-1, which further oligomerizes to convert pro-IL-1*β* and pro-IL-18 into bioactive peptides. (9) The biological significance of NLRP3 inflammasome activation is to release active IL-1*β* and IL-18 into extracellular space through exocytosis. The secreted IL-1*β* will facilitate inflammation process in the tissue whereas IL-18 will either promote caspase-3 dependent RPE apoptosis via MyD88 signaling or suppress neovascular vessels growth in the choroid capillaries.

The NLRP3 inflammasome senses and responds to a diversity of pathogen- or danger-associated molecular patterns (PAMPs or DAMPs), including bacterial/viral/fungal pathogens, pore-forming toxins, uric acid crystals, particulate aggregates, and adenosine triphosphate (ATP). To be activated, the NLRP3 inflammasome requires the presence of two signals, a “priming signal” and an “activation signal,” both of which are vital to control the degree of immune response driven by the products of inflammasome activation. In most cases, the “priming signal” channels through the nuclear factor kappa B (NF-*κ*B) pathway, upregulating the transcription of NLRP3 and prointerleukin-1*β* (pro-IL-1*β*) [37]. It is a fact that, in both immune and RPE cells, pro-IL-1*β* is not constitutively expressed and the endogenous level of NLRP3 appears to be inadequate for inflammasome activation, thus making the priming process critical [17, 38]. In contrast, other inflammasome-related proteins, ASC, procaspase-1, and pro-IL-18, are constitutively expressed in RPE cells and therefore priming may, or may not, further increase their protein levels [17, 20, 39, 40]. In addition to the classic, transcription-dependent priming, it is now known that NLRP3 inflammasome can be “primed” posttranslationally, adding another layer of regulation [41]. Common priming signals for immune cells of the body and human RPE cells are lipopolysaccharide (LPS), tumor necrosis factor-*α* (TNF-*α*), nitric oxide, and IL-1*α* [17, 42]. In the presence of foreign or endogenous “activation signals,” NLRP3 senses one or more of the following intracellular changes: K^+^ efflux [43], release of lysosomal resident cathepsin B [44], overproduction of reactive oxygen species (ROS) [45], NLRP3 translocation to mitochondria [46], cell volume change, and Ca^2+^ disequilibrium [42]. Once the NLRP3 is activated, it recruits ASC and mediates the proximity-induced procaspase-1 autoactivation. The assembled NLRP3 inflammasome then turns itself into a cytokine processing platform by cleaving pro-IL-1*β*/pro-IL-18 into mature peptides and releasing them into extracellular space for downstream effects (Figure 2). Of note, the generalized NLRP3 inflammasome activation process summarized above is much simpler than what actually happens in a given cell. Further discussion on NLRP3 inflammasome regulation will be provided in Section 4.

## 3. NLRP3 Inflammasome Activators Relevant to AMD

More recently, the role of the NLRP3 inflammasome in AMD pathogenesis has been extensively investigated using AMD related stimuli. Being a hallmark of AMD progression, drusen has a rich proteinaceous composition, including complement regulators, amyloid-beta (A*β*), and oxidation by-products [15, 47–54], which makes drusen components ideal triggers for potential interactions with the NLRP3 inflammasome.

### 3.1. Complement Factors

As part of the innate immunity, the complement system is one of the first that responds to tissue damage during aging and is activated by cell death [55]. In this regard, stressed, damaged, or dying RPE could trigger local complement activation, which is supported by the fact that activated complement components as well as complement regulators are present in drusen. The degree of complement activation is precisely regulated in healthy retina and is thus beneficial for tissue homeostasis and longevity. However, when the complement system is in overdrive due to either genetic polymorphisms [56] or chronic, sustained pathological stimulation, it generates undesirable amounts of activated complement factors, facilitates the formation of terminal membrane attack complex (MAC), and thus advances AMD pathology [57]. To bolster this notion, Doyle and colleagues have showed that drusen extracts isolated from AMD donor eye tissues are able to activate the NLRP3 inflammasome in LPS-primed macrophages [18]. They further revealed the role of complement factor 1q (C1q) as an NLRP3 inflammasome “activation signal” by showing caspase-1 cleavage and elevated IL-1*β* secretion after C1q stimulation on LPS-primed mouse bone marrow derived macrophages and THP1 human monocytic cells. Moreover, in addition to C1q stimulation, other studies have suggested complement factor 3a (C3a) and MAC may also mediate the activation of the inflammasome, further linking many components of the complement pathway with IL-1*β* and IL-18 production [24, 25, 58] by unique underlying mechanisms. For instance, sublytic MAC is known to activate NLRP3 inflammasome through Ca^2+^ influx and/or K^+^ efflux, whereas C3a activation of the NLRP3 inflammasome is initiated by the release of ATP into the extracellular space. These studies were primarily conducted on monocytes, dendritic cells, or lung epithelial cells. Although the aforementioned mechanisms are yet to be confirmed in AMD models, they do lend to the biological plausibility that similar processes can happen in ocular tissue. We recently tested the systemic administration of a small molecular inhibitor for MAC, aurintricarboxylic acid complex (ATAC), on NLRP3 inflammasome activation in rat RPE/choroid tissues at different ages. We found that ATAC suppressed the age-dependent increase in MAC formation and caspase-1 cleavage (a prominent indicator for NLRP3 inflammasome activation) in RPE/choroid tissue homogenates, which implies potential benefits by targeting MAC formation in AMD pathogenesis (Figure 2).

### 3.2. Amyloid-Beta

Amyloid-beta (A*β*) is a drusen component found in AMD eyes [51, 59, 60]; more recently there is growing interest in A*β* for its capacity to stimulate inflammasome activation and potentially contribute to AMD pathogenesis. As a pathological peptide best known for its neurotoxicity in Alzheimer's disease (AD), A*β* is generated through the amyloidogenic pathway by cleaving the amyloid precursor protein (APP) into the intramembrane A*β* domain of 36–43 amino acids in length [61]. The accumulation of A*β* in tissue results from its disturbed balance between production and clearance, the latter of which is largely controlled by the membrane-bound degradation enzyme, neprilysin [62, 63]. A*β*'s intrinsic cytotoxicity lies in its aggregated forms as soluble oligomers or insoluble fibrils. Originally thought as a primary toxic structure, A*β* fibrillar plaques are now considered less harmful to brain neurons than the small spherical oligomers that damage cell membranes and cause cell death [61, 64–68]. A*β*'s ocular presence has been reported in studies of postmortem human donor eyes [54, 60, 69] showing specific deposition within drusen from AMD eyes [53]. The age-dependent deposition of A*β* in the outer retina [59, 70] can be, at least partially, attributed to local RPE synthesis [51]. In this regard, transgenic animals lacking neprilysin exhibited A*β* accumulation in both RPE and sub-RPE deposits, concomitant with significant RPE atrophy [71]. When incubated with A*β* oligomers, human primary RPE cells demonstrated a prominent decrease in cell viability [72]. These findings point towards A*β*'s potential role in promoting RPE atrophy. Nevertheless, the exact mechanism by which A*β* contributes to RPE atrophy is still poorly understood but may involve inflammasome-related caspase-1 dependent cell death.

In addition to its cytotoxicity, A*β* is also a major proinflammatory factor that has been extensively studied in the context of AMD. The presence of A*β* in drusen is found to overlap with complement activation sites [47, 51]. In an RPE cell culture model, Kurji et al. discovered that inflammation-associated genes and immune response pathways were the predominant responses of RPE to oligomeric A*β* stimulation [72]. Proinflammatory responses to A*β* stimulation were also verified further in an in vivo model using intravitreal injections of A*β* in rodents, in which the RPE demonstrated upregulation of NLRP3, IL-1*β*, and IL-18 [69]. In this intravitreal A*β* injection model, inflammasome activation was demonstrated by caspase-1 cleavage, IL-1*β*, and IL-18 immunoreactivity in the RPE and surrounding tissue including the vitreous, which was further suppressed by an NF-*κ*B inhibitor, vinpocetine [19]. The secreted inflammasome effector cytokines, IL-1*β* and IL-18, are known to exert potent cytotoxic effects on RPE cells [40], which might provide a possible explanation for A*β* induced RPE cell death in vitro. These findings in RPE are consistent with a multitude of studies that implicate A*β* and NLRP3 inflammasome activation in glial cells and central nervous system disease, specifically AD. Using fibrillar A*β*, Halle et al. reported the activation of NLRP3 inflammasome by lysosomal destabilization which increased release of IL-1*β* in murine microglia [23] (Figure 2). To further substantiate the link between inflammasome activation and the deleterious effect of A*β* in the brain, APP/PS1 mice with NLRP3 deficiency demonstrated improved A*β* clearance by microglia and preserved memory and behavior patterns [73]. A*β*-induced NLRP3 inflammasome activation in glial cells may rely on activation of cathepsin family of proteases and the degradation of NLRP10 [74].

### 3.3. Oxidation By-Products

Having the most abundant polyunsaturated fatty acids in the eye, photoreceptors are protected by RPE cells from excessive high-energy light exposure. As part of the visual cycle, photoreceptors shed their oxidized tips of outer segments, which are then phagocytosed by RPE cells for the recycling of 11-cis-retinal. With age, the ability of RPE cells to recycle the “waste” from photoreceptors decreases significantly, leading to the accumulation of lipid peroxidation by-products, lipofuscin, in the RPE. It has been previously suggested that lipofuscin accumulation in RPE causes lysosome damage and directly triggers the formation of active NLRP3 inflammasome [17, 21, 75]. Other lipid peroxidation end products, such as 4-hydroxynonenal (HNE) and carboxyethylpyrrole (CEP), have also been shown to contribute to NLRP3 inflammasome activation [18, 22]. By incubating ARPE-19 cells, a cell line that possesses key features of human RPE cells, with HNE alone or in combination with LPS, Kauppinen et al. demonstrated a substantial increase of secreted IL-18 and IL-1*β*, products of NLRP3 inflammasome activation [22]. Doyle and colleagues discovered a priming effect for CEP, which promoted IL-1*β* secretion when combined with an inflammasome “activation signal,” such as ATP and C1q [18] (Figure 2). Furthermore, the recently established CEP-immunized murine model of AMD may serve as a useful platform to substantiate the role of CEP in NLRP3 inflammasome activation [76].

### 3.4. Genetic Variants

A disease with complex etiology, AMD, is also heavily influenced by genetic modifications. In GA patients, Kaneko et al. reported the repetitive element-derived* Alu* RNA transcripts as an inducer for RPE degeneration [77]. These retrotransposon elements are short interspersed nuclear elements in eukaryotic genome, containing internal promoter sequences for RNA polymerase III [78]. Thus, the* Alu* mobile repeats are noncanonical targets of DICER1, an evolutionarily conserved member of the RNase III nuclease family, essential for the control of microRNA biogenesis [79]. Originally considered as selfish “junk DNA” entities in the host genome, the* Alu* elements are now recognized for their complex regulatory functions, such as transcriptional repression [80] and modulation of alternative splicing [81], and involvement in human genetic diseases by inducing insertion mutations, DNA breaks, genome instability, and exonization [82, 83]. It has been shown that the loss of DICER1 expression, possibly due to oxidative stress in the RPE, is responsible for the abnormal* Alu* repeats accumulation in GA patients [77]. These* Alu* transcripts function as both priming (toll-like receptors independent, TLRs) and activating (P2X7 receptor dependent) signals to stimulate NLRP3 inflammasome activation, leading to the release of IL-18 and subsequent caspase-8/caspase-3 dependent apoptotic RPE death via MyD88 and/or Fas ligand mediated signaling [20, 40, 84]. These findings are novel as they provide us with a new perspective into the apoptotic RPE death mechanism underlying GA. These findings are also unique and counter intuitive at first appearance, since MyD88 is a versatile adaptor protein for the TLR/IL-1R superfamily-mediated proinflammatory signalling (reviewed in [85]), whereas apoptosis is essentially a noninflammatory cell death mechanism. Although very rare, there are cell-type specific reports connecting apoptosis with MyD88 mediated proinflammatory events, presumably as a final attempt for the tissue to remove the severely damaged cells. In pancreatic islet *β* cells, Dupraz et al. showed that overexpression of a dominant negative form of MyD88 spared the cells from IL-1*β* induced apoptosis [86]. Moreover, using human kidney epithelial 293 cells and human monocytic THP-1 cells, Aliprantis and colleagues found that MyD88 was crucial to the activation of TLR2 signaling and its downstream induction of caspase-8 dependent apoptosis in response to bacterial lipoproteins [87]. However, similar mechanisms have not been previously reported in ocular tissues or cells, including RPE, until recently.

Taken together, these lines of evidence suggest that damaged RPE cells can respond to danger signals by activating the inflammasome pathway that may further lead to RPE atrophy in GA (Figure 2).

## 4. Regulation of NLRP3 Inflammasome Activity

As a cellular property, the inflammasome is a powerful double-edged sword. Insufficient activation makes the immune system vulnerable to PAMPs and DAMPs, whereas overwhelming inflammasome activation targets the host itself. Hence, it is paramount to keep inflammasome activity in check. Clinically, inflammasome antagonists are being explored as novel therapeutics for treating human immune diseases [42, 88]. The inhibition of inflammasome can potentially be achieved at four different levels along its activation pathway [42]. These include blocking cell membrane receptors (e.g., P2X7 receptor for ATP), controlling cytoplasmic second messengers (e.g., K^+^, cathepsin B, ROS), preventing inflammasome components from assembling, and antagonizing released cytokine products and/or their cognate receptors. To better understand how inflammasome formation is regulated, we will review several newly discovered mechanisms.

### 4.1. Mitochondria and NLRP3 Inflammasome

Mitochondria, the symbolic “power house” in the cell, are also known for other biological events, including intrinsic or “mitochondrial” apoptosis and innate immune signal transduction [89]. The role of mitochondria in RPE dysfunction has previously been implicated in AMD pathogenesis [90]. In addition to lipid and protein peroxidation mentioned earlier in this review, mitochondrial DNA (mtDNA) is considered particularly prone to ROS relative to its nuclear counterpart. Research showed that when treated with hydrogen peroxide or rod outer segments human RPE cells generated more damaged and unrepaired mtDNA, leading to mitochondrial redox dysfunction, inefficient energy production, RPE dysfunction, and ultimate initiation of RPE apoptosis [90]. Moreover, it has been recently suggested that inherited mitochondrial DNA variation can also impact pathways other than apoptosis, for instance, in the complement activation [91]. By introducing mitochondria from individuals with either high- or low-risk haplogroups (accumulations of specific single nucleotide polymorphisms) for AMD into ARPE-19 cells devoid of mtDNA, Kenney et al. were able to map the relationship between mtDNA polymorphisms and nuclear gene expression for a number of molecular pathways related to AMD. ARPE-19 cybrids (cytoplasmic hybrids with native ARPE-19 cell nuclear DNA and extrinsic mtDNA) harboring high-risk mtDNA haplogroup for AMD demonstrated decreased energy production and lower gene expression levels for CFH and C3, key components of the complement pathway [92]. When confronted with sublethal ultraviolet radiation, these high-risk cybrids had a further decrease in CFH gene expression, which could potentially lead to a greater degree of complement activation [93]. As discussed in previous sections, complement activation products and MAC may act as potential triggers for inflammasome activation. However, the exact mechanisms bridging mitochondrial damage and RPE apoptosis remain elusive.

Bruey et al. reported a novel role of Bcl-2 and Bcl-xL, two mitochondria-associated antiapoptotic proteins, in the suppression of the NLRP1 inflammasome activation in ATP stimulated macrophages [94]. By comparing the macrophages isolated from Bcl-2 overexpressing transgenic mice to their wild-type counterparts, Zhou et al. showed a significant decrease of secreted IL-1*β* levels in association with the increasing Bcl-2 expression, under classic NLRP3 inflammasome activation conditions [45]. Separately, Shimada and colleagues also reported Bcl-2 attenuates NLRP3 inflammasome activity through inhibition of mtDNA release from the dysregulated and apoptotic mitochondria in macrophages [95]. However, another study using Bcl-2 overexpressing mice does not support Bcl-2's role in regulating NLRP3 inflammasome function [96]. Clearly, further investigation on the relationship between mitochondria-associated antiapoptotic proteins and NLRP3 inflammasome activation is warranted. Another example showcasing mitochondria's involvement in NLRP3 inflammasome activation comes from the studies on mitochondrial ROS, which has been proposed to be either necessary or facilitate the activation of the NLRP3 inflammasome, by two research groups, independently [45, 97]. Zhou et al. further proposed it was the dissociation between thioredoxin and thioredoxin-interacting protein (TXNIP) under ROS stimulation that allowed TXNIP to bind to NLRP3, resulting in NLRP3 conformational change and ultimate activation [98]. Moreover, the use of antioxidants to inhibit NLRP3 inflammasome activity has been extensively studied and proved effective, despite the fact that the detailed underlying signaling pathways remain unclear. Nonetheless, there is ongoing debate as to whether mitochondrial ROS only works as a facilitator of NLRP3 inflammasome activation, given the fact that increasing intracellular ROS levels can as well inhibit caspase-1 activation and IL-1*β* maturation [99–101]. Perhaps, a more comprehensive approach, such as a model reflecting intracellular antioxidant response to mitochondrial ROS overproduction, might provide insights regarding the determinants of the level of NLRP3 inflammasome activity, taking into account that both sides of the redox axis could play a role [102].

### 4.2. Ubiquitylation and Deubiquitylation

Ubiquitylation is a common posttranslational modification of proteins through a cascade of enzymatic activity of ubiquitin ligases. It has multiple effects on proteasome- or lysosome-mediated protein degradation, cell signal transduction, and protein activity regulation. According to the type and length of ubiquitin linkage, there are three major forms of protein ubiquitylation currently studied, including lysine 48 (K48) linked ubiquitylation, lysine 63 (K63) linked ubiquitylation, and methionine 1 (Met 1) linked linear ubiquitylation [103]. Mounting evidence has implicated the involvement of ubiquitylation in AMD pathophysiology. By looking at the retinal distribution of several class III ubiquitin-conjugating enzymes in mice, Mirza et al. reported robust protein expression of one such enzyme, UbcM2, in murine photoreceptors and RPE cells. The authors further experimented UbcM2's protective effects on photoreceptors using an acute bright-light-damage model. It was shown that mice with only one copy of functional UbcM2 allele were protected from acute excessive light damage to photoreceptors, suggesting a strong relationship between UbcM2-mediated ubiquitylation and photoreceptor survival [104]. In human RPE cells, it is evident that there is an active ubiquitin-proteasome mediated protein degradation pathway, with low endogenous levels of ubiquitin to fight against cellular stressors [105]. In the context of AMD, Ramos de Carvalhol et al. tested the proteasome activity of primary human RPE cells in response to complement factor C3a, a known drusen component. C3a stimulation significantly reduced proteasome activity without changing its component at either protein or mRNA levels, indicating a potential functional suppression of the proteasome in primary human RPE [106]. On the other hand, the family of deubiquitinating enzymes (DUBs) is another force balancing protein activity. Glenn and colleagues reported altered proteomic profiling of ARPE-19 cells cultured on advanced glycation end products (AGEs, known drusen component) modified Matrigel BM extract compared to non-AGE modified control BM. Of note, by immunocytochemistry, the authors were able to localize upregulated protein expression of a DUB protein, ubiquitin carboxyterminal hydrolase-1 (UCH-L1), in AGE-stimulated ARPE-19 cells, suggesting a potential role for DUBs in AMD pathogenesis [107].

However, few reports exist on the roles of both ubiquitinating and deubiquitinating enzymes in NLRP3 inflammasome regulation. Recently, Py and colleagues demonstrated that BRCC3, a JAMM domain-containing zinc metalloprotease DUB, promotes NLRP3 inflammasome activation by deubiquitinating the mixed K64 and K48 ubiquitin chains on both the NACHT and LRR domains of NLRP3 [26]. The authors further suggested that the deubiquitylation of NLRP3 was critical for the inflammasome activation based on the facts that inhibiting BRCC3 could abolish NLRP3 inflammasome activation under a diverse range of classic “activation signals,” including K^+^ efflux, ROS overproduction, and lysosomal destabilization. Perhaps more intriguing is the report by Rodgers et al. of the discovery that the linear ubiquitylation of the ASC adaptor protein by the linear ubiquitin assembly complex (LUBAC) is also essential for NLRP3 inflammasome activation, independent of NF-*κ*B activity [27] (Figure 2). Clinically, these studies provide potential alternative approaches for the treatment of inflammasome-related diseases by better controlling the ubiquitylation levels of separate NLRP3 inflammasome components, instead of targeting secreted levels of the mature proinflammatory cytokines.

## 5. Concluding Remarks

In this review, we summarize the key features of NLRP3 inflammasome activation and introduce newly discovered regulatory mechanisms. We also discuss the involvement of NLRP3 inflammasome in the pathogenesis of AMD, with the focus on individual drusen components as potential facilitators for NLRP3 inflammasome activation in RPE cells. Despite the rapid development of inflammasome research towards chronic inflammatory diseases such as AMD, there are still many unsolved questions. The biological significance of inflammasome activation in the outer retina remains controversial. On one hand, the mature product of inflammasome activation, IL-18, is hypothesized to carry out dual functions in different target cell types: as a destructive factor in GA [40] and as a protective, antiangiogenic factor in CNV [18]. On the other hand, the precise mechanisms underlying RPE demise in GA are still unclear. Does the NLRP3 inflammasome activation in RPE lead to canonical caspase-1 dependent pyroptosis [108] or necrosis or apoptosis or a combination of these mechanisms? It is well accepted that as AMD progresses, RPE cells follow a common fate: (1) accumulation of lipofuscin; (2) enlarged cell body; (3) decrease in phagocytosis capacity; (4) formation of drusen; (5) morphological rounding; (6) hyperpigmentation; (7) hypopigmentation; (8) RPE loss [109]. In sections of human postmortem donor eyes diagnosed with GA, Sarks et al. identified double-layered hyperpigmented RPE in the GA lesion, characteristic of necrosis [110], which was further supported by the discovery that RPE cells adjacent to excessive drusen accumulation die of necrosis [111]. Consistent with this description is the finding that necrosis, particularly the released ATP, is a trigger for NLRP3 inflammasome activation [112]. Further studies are needed to understand, more fully, the combination of cell death mechanisms associated with GA. The potential involvement of an apoptotic mechanism is also supported by several studies discussed earlier in this review and by a recent transcriptome analysis on AMD eyes [113]. Furthermore, much of our knowledge of the mechanisms associated with activation and regulation of the inflammasome comes from discoveries in nonocular immune cells. Validation of these mechanisms in ocular cell types, such as RPE and photoreceptors, will be useful towards designing inflammasome-related treatment strategies for chronic inflammatory diseases of the retina, such as AMD.
